# Turning C1-gases to isobutanol towards great environmental and economic sustainability via innovative biological routes: two birds with one stone

**DOI:** 10.1186/s13068-022-02202-1

**Published:** 2022-10-11

**Authors:** Bobo Liang, Rongzhan Fu, Yingqun Ma, Lizhen Hu, Qiang Fei, Xin-Hui Xing

**Affiliations:** 1 Shaanxi Key Laboratory of Energy Chemical Process Intensification, School of Chemical Engineering and Technology, Xi’an Jiaotong University, Xi’an, China; 2 Shaanxi Key Laboratory of Degradable Biomedical Materials, School of Chemical Engineering, Northwest University, Xi’an, 710069 China; 3 MOE Key Lab of Industrial Biocatalysis, Institute of Biochemical Engineering, Department of Chemical Engineering, Tsinghua University, Beijing, 100084 China

**Keywords:** Techno-economic analysis, Greenhouse gas, Bioconversion, C1-utilizing microbe, Sensitivity analysis, Isobutanol, Process design

## Abstract

**Background:**

The dramatic increase in greenhouse gas (GHG) emissions, which causes serious global environmental issues and severe climate changes, has become a global problem of concern in recent decades. Currently, native and/or non-native C1-utilizing microbes have been modified to be able to effectively convert C1-gases (biogas, natural gas, and CO_2_) into isobutanol via biological routes. Even though the current experimental results are satisfactory in lab-scale research, the techno-economic feasibility of C1 gas-derived isobutanol production at the industrial scale still needs to be analyzed and evaluated, which will be essential for the future industrialization of C1-gas bioconversion. Therefore, techno-economic analyses were conducted in this study with comparisons of capital cost (CAPEX), operating cost (OPEX), and minimum isobutanol selling price (MISP) derived from biogas (scenario #1), natural gas (scenario #2), and CO_2_ (scenario #3) with systematic economic assessment.

**Results:**

By calculating capital investments and necessary expenses, the highest CAPEX ($317 MM) and OPEX ($67 MM) were projected in scenario #1 and scenario #2, respectively. Because of the lower CAPEX and OPEX from scenario #3, the results revealed that bioconversion of CO_2_ into isobutanol temporally exhibited the best economic performance with an MISP of $1.38/kg isobutanol. Furthermore, a single sensitivity analysis with nine different parameters was carried out for the production of CO_2_-derived isobutanol. The annual plant capacity, gas utilization rate, and substrate cost are the three most important economic-driving forces on the MISP of CO_2_-derived isobutanol. Finally, a multiple-point sensitivity analysis considering all five parameters simultaneously was performed using ideal targets, which presented the lowest MISP of $0.99/kg in a long-term case study.

**Conclusions:**

This study provides a comprehensive assessment of the bioconversion of C1-gases into isobutanol in terms of the bioprocess design, mass/energy calculation, capital investment, operating expense, sensitivity analysis, and minimum selling price. Compared with isobutanol derived from biogas and natural gas, the CO_2_-based isobutanol showed better economic feasibility. A market competitive isobutanol derived from CO_2_ is predicable with lower CO_2_ cost, better isobutanol titer, and higher annual capacity. This study will help researchers and decision-makers explore innovative and effective approaches to neutralizing GHGs and focus on key economic-driving forces to improve techno-economic performance.

## Background

As a building block chemical, isobutanol has been widely applied in various fields, including food, solvents, extractants, rubber, fuel additions, and transportation fuels [1–3]. At present, isobutanol is generally produced via a chemical route under harsh conditions [4, 5]. Recently, the biological route has been widely considered as an environmentally friendly approach for isobutanol synthesis with sugar-based substrates, which may not be sustainable, as it inevitably compromises the future food supply [6, 7]. Therefore, it is urgently needed to explore alternative inedible and abundant carbon sources, such as greenhouse gas (GHG), that are consistent with the best interest of global sustainability [8]. CO_2_ and CH_4_ are two major GHGs causing the global warming effect, which is one of the greatest environmental problems in the world. These two C1-gases can be derived from fossil fuels [9] and anaerobic digestion (AD) [10, 11] in abundant amounts [12–16]. This in turn suggests that these gases have a large potential to be a promising carbon source for C1-utilizing microbes. It has been considered that biological valorization of C1-gases into isobutanol could be a promising route giving great environmental and economic sustainability as well as reduction of GHG emissions as two birds with one stone.

**Fig. 1:**
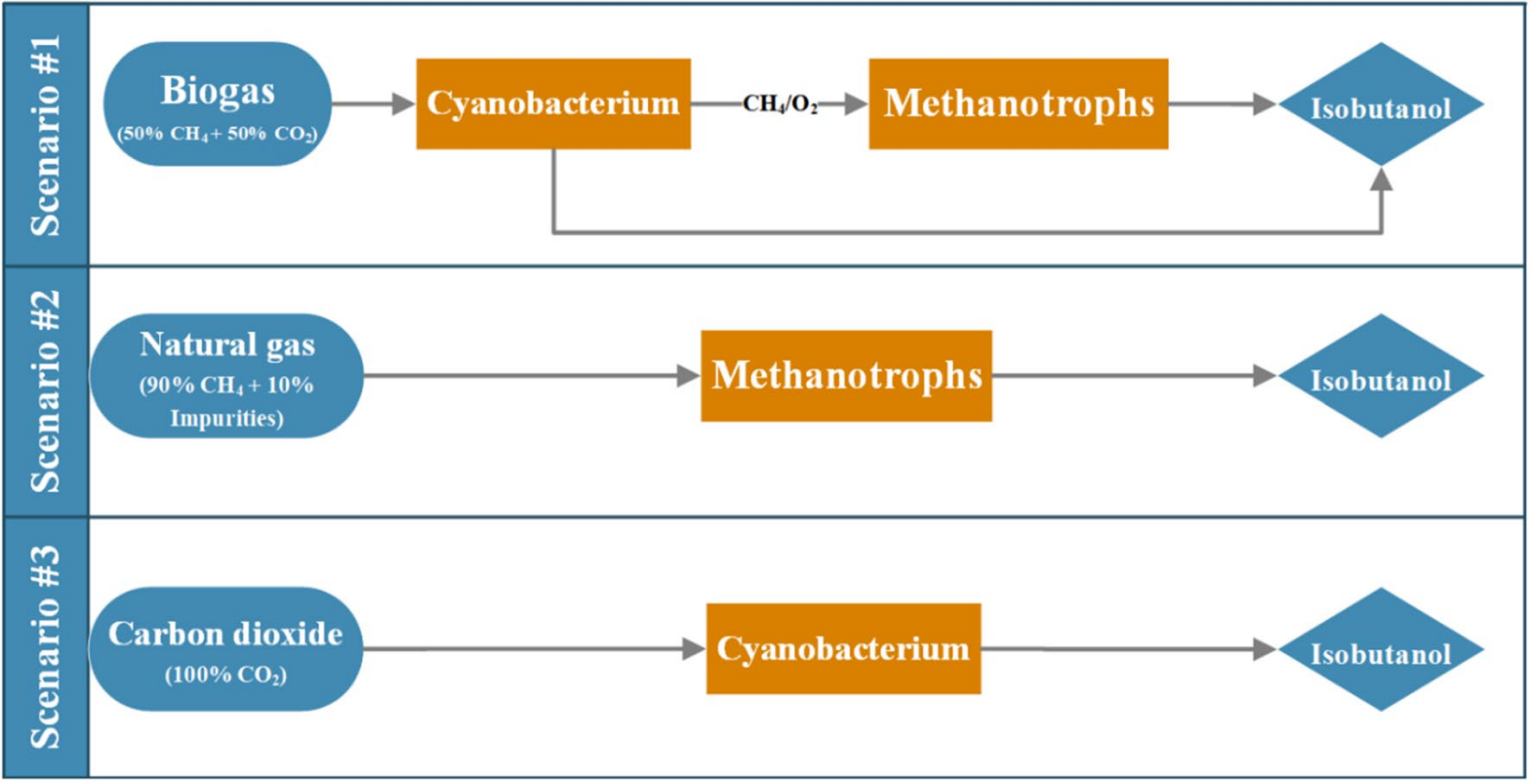
Process scenarios of isobutanol production by different microorganisms using various C1 gases. Scenario #1: isobutanol production from biogas; scenario #2: isobutanol production from natural gas; scenario #3: isobutanol production from CO_2_

Currently, more efforts have been made to convert these abundant and low-cost C1-gaseous substrates into isobutanol by biological routes and systems engineering of C1-utilizing microbes [17], as shown in Fig. 1. More than 900 mg/L isobutanol has been demonstrated by using CO_2_ as the sole carbon source in the autotrophic cultivation of *Synechocystis PCC* 6803 [18]. In recent years, native and/or non-native C1-utilizing microbes have been modified or constructed by using genetic engineering tools, systematic manipulation, metabolic modeling, and carbon flux simulation to improve the C1-gas utilization rate [19, 20]. However, the challenges and opportunities for methane bioconversion into isobutanol by methanotrophs remain in both scientific and industrial applications. Although Precigen Inc. (formerly named Intrexon Corporation), a biosynthesis-based company, has claimed that CH_4_-derived isobutanol has been accomplished on a lab scale by engineered methanotrophic bacteria [21], no scientific results have been published thus far. Therefore, to verify the possibility of isobutanol biosynthesis from CH_4_, *Methylomicrobium buryatense* (an industrially proven methanotrophic bacteria) was genetically engineered to achieve direct conversion of CH_4_ into isobutanol in our laboratory. By heterologously expressing α-ketoisovalerate decarboxylase (Kivd) derived from *Lactococcus lactis*, isobutanol-producing *M. buryatense* was constructed, which accumulated approximately 35 mg/L in 5 days under unoptimized conditions in vials with a very limited gas transfer efficiency [22, 23]. It has been reported that a 1000-fold increase in isobutanol productivity can be achieved by using C1-gaseous substrates in airlift bioreactors [24–26]. In addition, the carbon conversion efficiency can also be significantly improved by genetically engineering key enzymes for isobutanol biosynthesis [18, 27]. Recently, a biotech pioneer company, LanzaTech, successfully scaled up a gas fermentation process to convert low-cost waste gas feedstocks into isopropanol using engineered autotrophic acetogen [28]. In that study, isopropanol productivity was enhanced from 0.6 g/L/h up to 3 g/L/h with optimizations in both strain construction and process development. Although the bioconversion of CO_2_ or CH_4_ into isobutanol has not been reported on a large scale, it is highly predictable that industrial applications of CO_2_/CH_4_-based isobutanol production will be achieved with the rapid development in synthetic biology technologies and processes.

Although the current experimental results are satisfactory in lab-scale research, the economic feasibility and effective applications at the industrial scale still need to be fully analyzed and evaluated. Therefore, a techno-economic analysis (TEA) was projected in this paper, which is a framework for quantitatively evaluating the economic performance of a specific process design based on published bioconversion and process integration research from the aspects of cost, benefit, risk and uncertainty, aiming to evaluate its future commercial potential and guide research and investment in the most beneficial direction [29]. The goal of this TEA study is to evaluate the economic potential of the three designed scenarios of the bioconversion of biogas, natural gas, and CO_2_ to isobutanol with comparisons of capital cost, operating cost, and minimum isobutanol selling price (MISP). Finally, single- and multiple-point sensitivity analyses were also carried out to guide the engineering practice of isobutanol production with the most economic potential. This study will help researchers and decision-makers focus on key economic-driving forces to improve the techno-economic performance of the bioconversion of C1-gases for isobutanol production.

### Process designs and assumptions for isobutanol production from C1 gaseous substrates

#### Scenarios

To evaluate the technical and economic performances of isobutanol production from biogas, natural gas, and CO_2_, the following three bioroutes were designed and designated scenario #1, scenario #2, and scenario #3, as shown in Fig. 1. For scenario #1, both CH_4_ and CO_2_ in biogas are converted into isobutanol directly in a two-stage cultivation by cyanobacteria and methanotrophs, respectively. Natural gas and CO_2_ are used as the sole carbon sources for isobutanol biosynthesis in scenario #2 and scenario #3, respectively. The simulation was accomplished by commercial process software (AspenTech^®^, Cambridge, MA, USA) to obtain rigorous material and energy balances for each unit operation.

### Process design

**Fig. 2:**
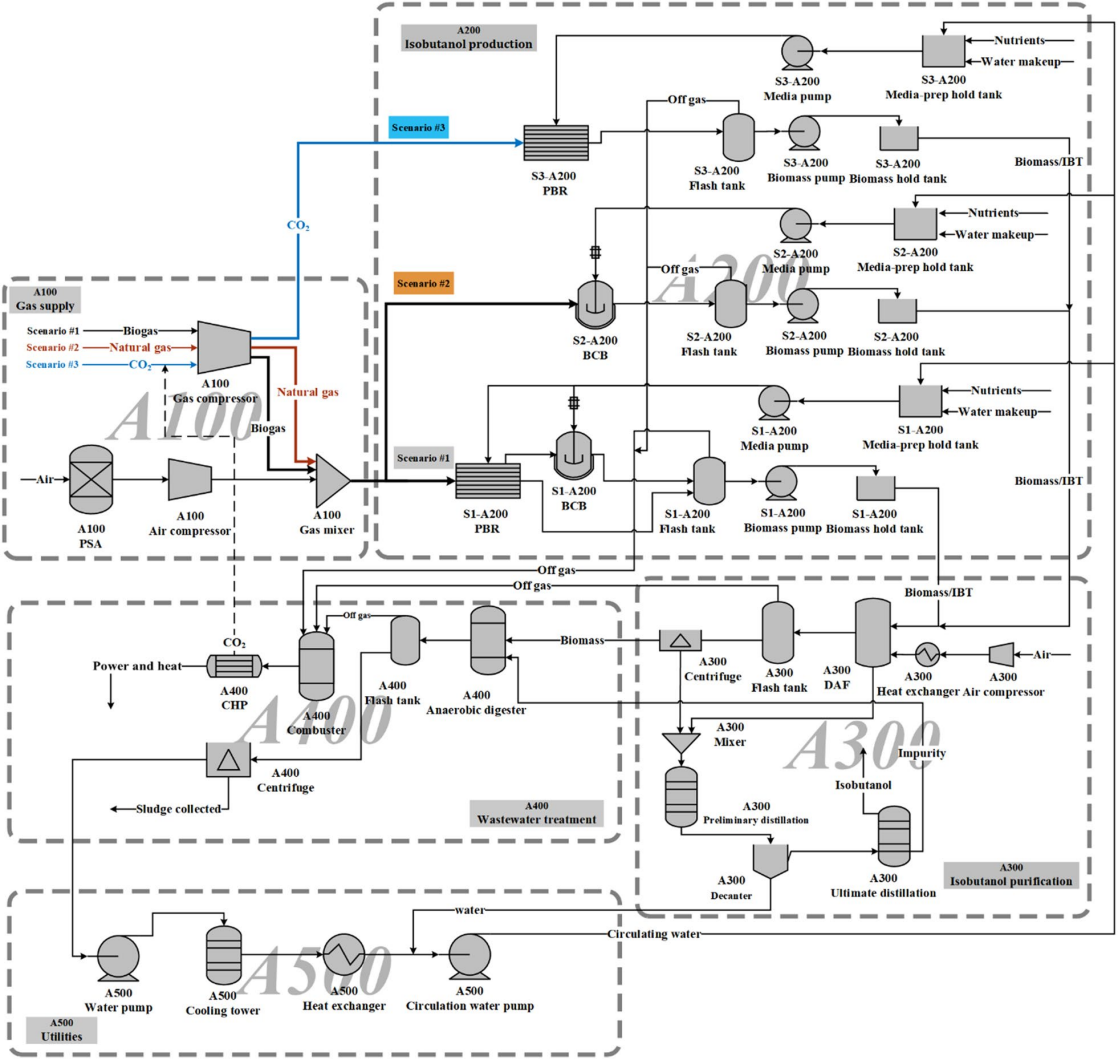
Schematic process flow diagram of isobutanol production from C1 gases. A100: gas supply; A200: isobutanol production; A300: isobutanol purification; A400: wastewater treatment; A500: utilities; PBR: closed tubular photobioreactors just for culturing autotrophic microbes using CO_2_; BCB: bubble column bioreactors just for culturing heterotrophic microbes using CH_4_

As shown in Fig. 2, a simplified flow diagram is composed of five sections: gas supply, isobutanol production, isobutanol purification, wastewater treatment, and utilities. The mass and energy flow balance in Aspen simulation models was calculated based on a *n*th isobutanol plant with an annual production capacity of 50,000 tons. TEA was carried out in the sequence of schematic design, process simulation and economic evaluation. The parameters related to the TEA, including operating, financing, and cost investments, are all summarized in Table 1 [16]. These data will be manipulated on an Excel spreadsheet that incorporates well-established formulas to determine the total capital investment (TCI) and total operating cost (TOC) of designed processes [29]. A 10-year discounted cash flow rate of return analysis was used to estimate the MISP at a net present value of zero with 10% internal rate of return (IRR) “*n*th-plant” costs and financing.

**Table 1:** Assumptions for *n*th-plant isobutanol production

Description of assumption	Value
Internal rate of return (IRR)	10%
Plant financing by equity	50%
Plant life	30 years
Income tax rate	21%
Interest rate for debt financing	8%
Term for debt financing	10 years
Working capital cost	5% of fixed cost investment (FCI)
Land purchase cost	1% of fixed cost investment (FCI)
Depreciation schedule	7-year MACRS schedule
Start-up time	6 months
Revenue and cost during startup	Revenue = 50% of normal Variable costs = 75% of normal Fixed costs = 100% of normal
Operating hours per year	7920

#### Gas supply (area 100)

In this area, remote C1-gaseous substrates, such as biogas, natural gas, or CO_2,_ are compressed to the ideal pressure of 5 bar [30] to satisfy the actual fermentation conditions and avoid the pressure fluctuation of the reaction system, which is then sent to bioreactors for isobutanol production. Although the biogas composition varies with the feedstock during the AD process, the ratio of CH_4_ and CO_2_ in biogas used in scenario #1 was assumed to be 1:1. Natural gas containing 90% CH_4_ and 10% harmful impurities on methanotroph growth was used in scenario #2 [31]. The substrate used in scenario #3 was assumed to be 100% CO_2_. The air stream routed to a pressure swing adsorption (PSA) unit is enriched into 95% O_2_ for methanotrophic cultivation in scenario #1 and scenario #2. The design specification tool in Aspen was used to obtain required amounts for different gas feeds at a specific production scale for calculations in other processes in this study. To achieve an annual production (plant capacity) of 50,000 tons isobutanol, the required amounts of gaseous substrates (oxygen not included) were individually calculated as 388,740 m^3^/day, 278,250 m^3^/day, and 321,338 m^3^/day for scenario #1, scenario #2, and scenario #3 according to Eqs. 3 and 4 in Aspen, respectively.

#### Isobutanol production (area 200)

The isobutanol production process involves both biomass generation and isobutanol biosynthesis by either cyanobacteria cultured in a series of closed tubular photobioreactors (PBRs) with a 50 m^3^ working volume [32] or methanotrophs grown in several 1000 m^3^ bubble column bioreactors (BCBs) with an 80% working volume [16, 33]. The stoichiometry equations for biomass generation by cyanobacteria (Eq. 1) and methanotrophs (Eq. 2) applied in Aspen simulations have been proposed based upon published literature [34, 35], in which the empirical formula CH_1.934_O_0.473_N_0.23_ and C_4_H_8_O_2_N represent the biomass from cyanobacteria and methanotrophs, respectively. The stoichiometry equation for isobutanol biosynthesis in cyanobacteria and methanotrophs has been assumed according to the published report as Eq. 3 and Eq. 4, respectively [5, 18]. For scenario #1, the biogas is first sent into the PBRs, where CO_2_ can be immobilized by cyanobacteria via photosynthesis for isobutanol biosynthesis and O_2_ production. The off-gases from PBRs containing CH_4_, O_2_, and unused CO_2_ are transferred to BCBs for isobutanol production by aerobic-obligate methanotrophs.

1 $${\text{CO}}_{{2}} { + 0}{\text{.622H}}_{{2}} {\text{O + 0}}{\text{.23NH}}_{{3}} \to {\text{CH}}_{{{1}{\text{.934}}}} {\text{O}}_{{{0}{\text{.473}}}} {\text{N}}_{{{0}{\text{.23}}}} { + 1}{\text{.0745O}}_{{2}}$$

2 $${\text{CH}}_{{4}} { + 1}{\text{.5O}}_{{2}} { + 0}{\text{.118NH}}_{{3}} \to {0}{\text{.118C}}_{{4}} {\text{H}}_{{8}} {\text{O}}_{{2}} {\text{N + 0}}{\text{.529CO}}_{{2}} { + 1}{\text{.71H}}_{{2}} {\text{O}}$$

3 $${\text{4CO}}_{{2}} {\text{ + 5H}}_{{2}} {\text{O}} \to {\text{C}}_{{4}} {\text{H}}_{{{10}}} {\text{O + 6O}}_{{2}}$$

4 $$6{\text{CH}}_{{4}} {\text{ + 6O}}_{{2}} \to {\text{C}}_{{4}} {\text{H}}_{{{10}}} {\text{O + 2CO}}_{{2}} {\text{ + 7H}}_{{2}} {\text{O}}$$

Since carbon and nitrogen sources account for most of the raw material cost [36], only gaseous substrate and ammonia were considered for the economic analysis in this study. The usage of these raw materials could be calculated based on the aforementioned equations by Aspen software for operation cost. The isobutanol productivity of 1 g/L/h is the minimum productivity required for industrial applications of biomanufacturing, and the highest isobutanol titer of 1 g/L reported in the literature [18] was assumed to be the baseline value of the Aspen simulation in all three scenarios. By using the productivity and stoichiometry equations (Eqs. 3 and 4) for isobutanol biosynthesis, the total fermentation volume of scenarios #1, #2, and #3 was calculated as 10,059 m^3^, 10,120 m^3^, and 10,305 m^3^, respectively, which requires 126 bioreactors including 121 PBRs and 5 BCBs for scenario #1, 13 BCBs for scenario #2, and 206 PBRs for scenario #3. The highest utilization rate of gaseous substrates was observed as high as 90% in both PBR [37, 38] and BCB [39], which were applied in our TEA. As an extracellular product, isobutanol production is not associated with cell growth, and 20% of gaseous substrate supply was simulated to be used for cell growth based on carbon-balance, and the rest for isobutanol biosynthesis [40–42].

#### Isobutanol purification (area 300)

The effluent from A200 is first pretreated with a three-step dewatering process to separate the liquid phase and cell mass. A dewatering system containing decanting, dissolved air flocculation (DAF) and centrifuge was applied in this area. After decanting, a DAF separator with flocculants is followed by a solid/liquid separation step using centrifugation [43, 44]. The isobutanol–water mixture obtained from the dewatering process will be further purified using two distillation columns and one decanter to harvest isobutanol from the liquid phase [45]. In detail, the first column concentrates the overhead product by removing water, high boilers (organic acids) and solids to the ratio of isobutanol and water for a liquid–liquid split. The isobutanol-rich stream is sent to the second column for high purity isobutanol as the final product. The overall separation efficiency of this process is up to 90%, which was set in the Aspen simulation.

#### Wastewater treatment (area 400)

As shown in Fig. 2, all effluents collected from A300 composed of wastewater, spent cell mass, and other liquid streams are directed to an on-site AD plant. The biogas generated from AD along with other gases collected from A200 is transferred to combustion facilities for on-site energy generation [43]. A large amount of high-nitrogen sludge extracted from AD was assumed to be recovered as a byproduct for fertilization [46].

#### Utilities (area 500)

Area 500 facilitates overall energy, water, and power integration, including a cooling water system, chilled water system, process water manifold, and power systems. In this area, the amount of make-up water required in the process could be determined as well as the total power requirements for the system and electricity purchased from the grid.

### Process economics analysis

This study aims to assess and analyze the economic feasibility and competitiveness of various integrated processes of isobutanol production from C1-gaseous substrates to guide practical engineering activities. It is worth mentioning that all methods or data used in this TEA are obtained from published literature and official reports [16, 29, 47].

#### Capital expenses (CAPEX) and discounted cash flow method

The capital expenses were calculated by considering the total cost of equipment purchases and associated installation. The costs of units or equipment used in this study are obtained from previous studies, as listed in Table 2, and the new equipment costs for different sizes were calculated using the exponential scaling expression (New cost = Base cost × (New size/Base size)^*n*^, where *n* is the economy scaling factor and varies with the equipment) based on the equipment size for the original price quote [21]. The Chemical Engineering Plant Cost Index (CEPCI) [48] was used to recalculate equipment costs to 2020 dollar (2020$) [29]. The assumptions used for this TEA can be found in Table 1, which were proposed based on previous literature [49, 50].

**Table 2:** Major equipment investments for three scenarios with an annual plant capacity of 50,000 tons

Area	Equipment	Installed cost, MM$			References
		Scenario #1	Scenario #2	Scenario #3	
100	Gas compressor package	0.31	0.53	0.27	[33]
	Heat exchangers	0.06	0.13	0.04	[44]
	Air separation unit	1.32	7.84	0.00	[58]
200	Pump	4.08	3.37	3.42	[49]
	Media-prep tank	49.99	38.53	39.05	[49]
	Media-prep tank agitator	0.60	0.40	0.41	[49]
	Closed tubular photobioreactors	0.59	0.00	0.94	[32]
	Bubble column seed bioreactor	0.80	1.18	0.00	[33]
	Bubble column bioreactor	17.84	36.25	0.00	[33]
	Fermentation transfer pump	0.15	0.26	0.00	[33]
	Flash tank	10.75	14.54	14.71	[33]
300	Dissolved air flotation separator	6.96	6.60	6.71	[49]
	Centrifuge	4.81	4.65	4.71	[49]
	Distillation feed pump	4.09	3.93	3.90	[49]
	Distillation column	105.67	100.92	100.32	[33]
	Decanter	2.57	2.46	2.45	[33]
400	Anaerobic digester systems	1.91	0.12	1.82	[44]
	Conveyor	1.52	0.04	1.46	[49]
	Combustor	0.15	0.31	1.37	[44]
	Heat exchangers	0.33	0.36	0.19	[44]
	Flash tanks	15.42	14.54	14.68	[33]
500	Cooling tower system	2.30	2.16	2.18	[33]
	Cooling water pump	0.73	0.69	0.70	[33]
	Make-up water pump	0.58	0.55	0.02	[33]
	Heat exchangers	4.43	4.19	4.23	[44]

#### Operating expenses (OPEX)

The OPEX, including gaseous substrates, other raw materials, and fixed operating costs, are depicted in Table 3. In this study, all values derived from published TEA reports or literature have been converted into 2020$ based on the Industrial Inorganic Chemical Index from SRI Consulting for raw materials [51] and the labor indices from the US Department of Labor Bureau of Labor Statistics for employee salaries [52]. The labor burden was estimated to be 60% of the total wage, and 2% of the inside boundary limit (ISBL) capital expenses were designated for maintenance. In addition, local property tax and property insurance were estimated at 0.5% of fixed capital investment [53].

**Table 3:** Costs of raw materials used in the base case study

Raw materials	Cost	Unit	References
Inputs			
Biogas	130.3	$/ton	[59]
Carbon dioxide	74.1	$/ton	[60]
Natural gas	182.5	$/ton	[61]
Ammonia	431	$/ton	[33]
Flocculant	9670	$/ton	[58]
Electricity	0.1	$/KW	[62]
Water	0.2	$/ton	[63]
Cooling tower chemicals	3372	$/ton	[49]
Sludge disposal cost	15.9	$/ton	[64]
Outputs			
Electricity credit	0.1	$/KW	[62]
CO_2_ credit	30.2	$/ton	[65]
AD sludge N credit	271	$/ton	[44]

## Economic comparison of isobutanol production from C1 gaseous substrates

### Comparison of CAPEX and OPEX

**Fig. 3:**
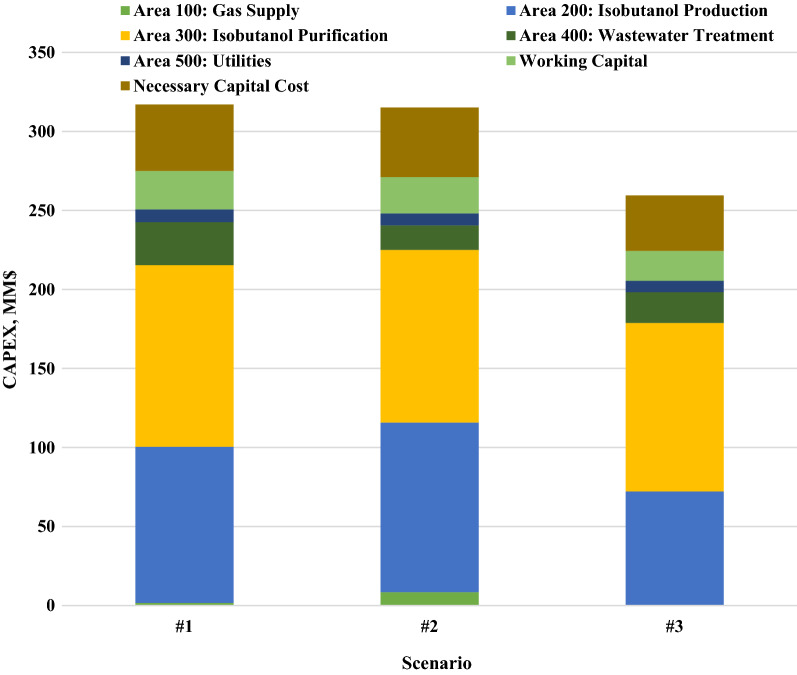
Capital cost distributions of three scenarios with an annual plant capacity of 50,000 tons. Scenario #1: isobutanol production from biogas; scenario #2: isobutanol production from natural gas; scenario #3: isobutanol production from CO_2_. Necessary capital cost refers to the sum cost of warehouse, site development, additional piping and land

To explore the most economic potential scenario, a detailed comparison of capital investments was obtained by using chemical engineering cost estimation techniques, as shown in Fig. 3, in which the necessary capital cost refers to the capital costs of warehouse, site development, additional piping and land. A similar CAPEX was observed in scenario #1 ($317 MM) and scenario #2 ($315 MM), which are relatively higher than that in scenario #3 ($259 MM). This is attributed to the lower equipment investment of A100 and A200 in scenario #3. Due to the high cost of traditional BCBs used in methanotrophic cultivation in scenarios #1 and #2 (Table 2), a total CAPEX reduction of more than 17% can be achieved in scenario #3, profiting from the low cost of PBRs for cyanobacteria cultivation. It should also be noted that the major difference in CAPEX among the three scenarios is that scenario #3 does not require BCBs, while the investment in BCBs purchases in scenarios #1 and #2 is more than $36 MM and $17 MM, respectively. Table 4 showed that the OPEX of the three scenarios presents the major difference in the cost of carbon and nitrogen sources. Scenario #3 has the lowest total operating cost of $55.09 MM due to the relatively low cost of CO_2_. Interestingly, the C1-gasous substrates in all three scenarios only contribute approximately 30–40% of the total OPEX, which is significantly lower than the proportion (60–80%) of conventional raw materials (e.g., glucose) and may improve the economic viability and market potential using C1-gasous substrates as carbon sources for isobutanol production. It is worth noting that the differences in cost for the three different scenarios are also caused by the sourcing of gases, equipment manufacturing or plant location. Novel bioreactor designs for CH_4_ or CO_2_ bioconversion may reduce both CAPEX and OPEX.

**Table 4:** Comparisons of operating costs in three scenarios with an annual plant capacity of 50,000 tons

Manufacturing costs			
Item	Annual cost (MM$/year)		
Variable operating costs-raw materials	Scenario #1	Scenario #2	Scenario #3
Gaseous feedstock	24.71	30.32	17.61
Ammonia	2.41	2.28	3.24
Flocculant	12.75	12.84	13.05
Sludge disposal cost	13.13	14.30	14.44
Makeup water	0.18	0.15	0.20
Cooling tower chems	0.06	0.06	0.06
Electricity	0.00	2.34	0.00
Sum of variable operating cost of raw materials	53.23	62.29	48.60
Variable operating costs-byproduct credits			
AD sludge *N* credit	1.13	0.96	1.27
CO_2_ credit	1.35	4.77	0.00
Grid electricity	0.82	0.00	1.44
Sum of byproduct credits	3.30	5.73	2.71
Total variable operating costs (VOC)	49.93	56.56	45.89
Fixed operating costs			
Salaries	2.33	2.33	2.33
Labor burden	1.40	1.40	1.40
Facility maintenance	4.00	4.50	3.58
Property insurance and tax	2.29	2.30	1.89
Sum of fixed operating costs (FOC)	10.02	10.53	9.20
Total operating cost (VOC + FOC)	59.95	67.09	55.09

### Minimum isobutanol selling price (MISP) from different scenarios

Determining a minimum selling price based on investment expense refers to analyzing the cost of a business decision in terms of the real-time relevant expenses. The minimum pricing is the break-even point for that given sale. According to the global isobutanol production market, the *n*th plant with an annual capacity of 50,000 tons isobutanol is projected, and the resulting MISP of the three scenarios at a 10% IRR is $1.51/kg isobutanol, $1.81/kg isobutanol, and $1.38/kg isobutanol in scenarios #1, #2, and #3, respectively. Apparently, because of the lowest CAPEX and OPEX, the most inexpensive MISP of $1.38/kg isobutanol is obtained in scenario #3, which is 10% and 20% lower than the MISP from scenario #1 and scenario #2, respectively. Given the state of the art, CO_2_-derived isobutanol is projected to be the best scenario by assuming 90% utilization efficiency of CO_2_. Given the promising economic feasibility of scenario #3, sensitivity analyses of the bioconversion of CO_2_ to isobutanol are also carried out to investigate the impact of key variables on MISP and to consider the options for optimizing process economics.

## Sensitivity analysis for isobutanol production from CO_2_

### Single-point sensitivity analysis

Sensitivity analysis is an efficient approach to quantify the impact of key variables on the overall MISP. Therefore, a single-point sensitivity analysis is performed on the isobutanol production process using CO_2_ by adjusting only one single variable between its minimum and maximum value with all others kept constant. In this study, nine variables associated with CO_2_-derived isobutanol production were evaluated for their influence on MISP. The baseline for all variables was the same as the assumptions used in the case of scenario #3. For the sensitivity analysis, a "Calculator" module in Aspen was applied to manually adjust these variables, and then by running the simulations in Aspen, the required values such as reaction volume, medium dosage, etc., under different production requirements were projected by Aspen software for calculating MISP.

**Fig. 4:**
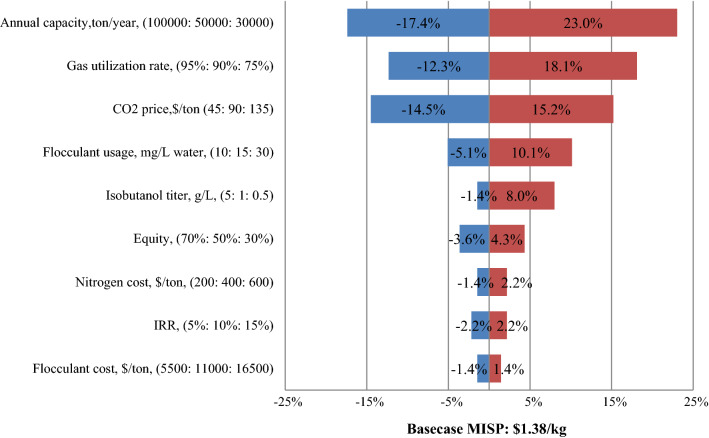
Single-point sensitivity analysis of the minimum selling price of isobutanol (MISP) from CO_2_

As shown in Fig. 4, the annual plant capacity, gas utilization rate, CO_2_ price, flocculant dosage, and isobutanol titer have the largest impact on the MISP with a nearly 10% variation, which are key cost drivers. The annual plant capacity, gas utilization rate, and isobutanol titer are negatively correlated with MISP, whereas CO_2_ price and flocculant dosage are positively correlated. The plant capacity is directly associated with the gas supply and flow rate, thus affecting CAPX, OPEX and MISP. It can also be observed that the MISP can be finally reduced from $1.38/kg to $1.14/kg when the plant capacity doubled from 50,000 to 100,000 ton/year, resulting in an increase in the flow rate from 321,338 to 726,030 m^3^/day simulated in Aspen.

Following the annual capacity, the gas utilization rate is the second most important cost-driving force impacting MISP, which will boost MISP up to 120% if the utilization rate is decreased by 25%. This finding might be attributed to the fact that the utilization rate strongly affects the isobutanol yield, which in turn leads to a significant impact on raw material expenses and bioreactor investment. It is well known that the cost of glucose used in a bioprocess may contribute up to 60% of the total raw material costs [54], even if shell corn is directly used as raw material, the cost will also account for more than 45% [16, 45], while the cost of CO_2_ in scenario #3 only accounts for 36% of the cost of raw materials (Table 4), which dramatically reduces the MISP. It can be expected that using wasted CO_2_ collected from the concrete industry or power plants could further decrease the production cost [55]. In addition to the aforementioned variables, other parameters selected in this study (such as equity, nitrogen price and IRR) show minimal contributions to MISP. Based on the findings from single-point sensitivity analysis, key cost drivers with optimal values were chosen as targeted goals in the multiple-point sensitivity analysis for a long-term case.

### Multiple-point sensitivity analysis

**Fig. 5:**
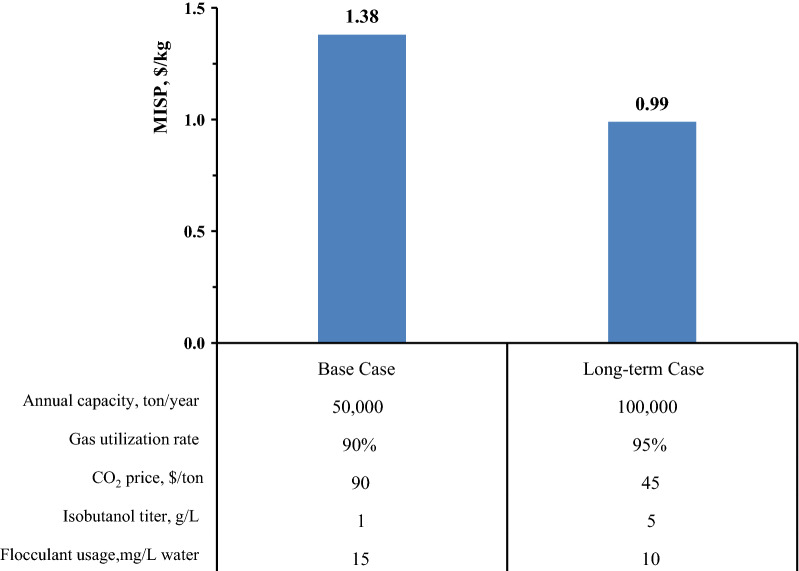
Multiple-point sensitivity analysis of various prospective targets on the minimum selling price of isobutanol (MISP) from CO_2_

To obtain a comprehensive understanding of the effects of key factors on the economic performance of CO_2_-derived isobutanol, an exhaustive sensitivity analysis was conducted by simultaneously adjusting five key variables for MISP calculation. As shown in Fig. 5, two situations, including the base case (baseline in the single-point sensitivity analysis) and long-term case, were illustrated and compared to project the cost potentials of the proposed technology pathway. Given the optimization of genetic engineering, enhancement of the bioconversion process, and improvement of CO_2_ capture efficiency [56], the isobutanol concentration, annual production, and CO_2_ price were rationally predicted. The MISP for the long-term case can be as low as 0.99 $/kg isobutanol by using ideal targets. This MISP is close to the current market price of $1.0/kg isobutanol [57], which in turn shows the both environmental and economic potential of CO_2_-based isobutanol considering the carbon tax policy and GHG emission reduction. Although currently demonstrated technology and recently published results of isobutanol biosynthesis from CO_2_ are still far from our ideal targets, it is believed that the advanced biotechnologies can fill in the gap by constructing genetically engineered microorganisms with higher carbon yield and faster growth rate, reaching theoretical conversion efficiency, and having minimum raw material cost.

## Conclusions and prospective

In this study, TEA was applied to calculate the OPEX and CPAEX of the proposed biological routes to evaluate the economic feasibility and industrialization potential of the bioconversion of C1-gaseous substrates for isobutanol production. With the lowest OPEX and CAPEX, the CO_2_-derived isobutanol presents the lowest MISP of $1.38/kg isobutanol compared with that derived from biogas and natural gas. By employing single/multiple-point sensitivity analyses, the annual plant capacity, gas utilization rate, and CO_2_ price are determined to be key cost drivers. With the expected research targets, the promising MISP of $0.99/kg isobutanol can be achieved by reducing CO_2_ cost and enhancing the production performance of isobutanol production. Currently, biogas, natural gas, and CO_2_ are the main sources of GHG emissions, of which the amounts are projected to further increase in the coming decades. By adopting the proposed biological routes, the C1-gases, including biogas, natural gas, and CO_2,_ can be used as an alternative inedible substrate for isobutanol production, which is consistent with the best interest of global environmental and economic sustainability. It is expected that this study may provide engineering practice guidance and cost optimization strategies for the future biological conversion of C1 greenhouse gases to platform chemicals.

## Data Availability

Not applicable.

## Abbreviations

GHGs: Greenhouse gases
TEA: Techno-economic analysis
MISP: Minimum isobutanol selling prices
R&D: Research and development
CCE: Carbon conversion efficiency
IRR: Internal rate of return
CAPEX: Capital expenses
OPEX: Operating expenses
AGR: Amine-based acid gas removal
ASU: Air separation unit
CSTR: Continuous stirred tank reactors
DAF: Dissolved air flotation
AD: Anaerobic digestion
ISBL: Inside battery limit
FCI: Fixed cost investment
SOT: State of technology
LCA: Life cycle assessment
TCI: Total capital investment
TDC: Total direct costs
TIC: Total indirect costs
TOC: Total operating cost
GHG: Greenhouse gas
GWP: Global warming potential
